# Shape information from glucose curves: Functional data analysis compared with traditional summary measures

**DOI:** 10.1186/1471-2288-13-6

**Published:** 2013-01-17

**Authors:** Kathrine Frey Frøslie, Jo Røislien, Elisabeth Qvigstad, Kristin Godang, Jens Bollerslev, Nanna Voldner, Tore Henriksen, Marit B Veierød

**Affiliations:** 1 Department of Biostatistics, Institute of Basic Medical Sciences, University of Oslo, Boks 1122, Blindern, 0317, Oslo, Norway; 2 Norwegian Resource Centre for Women's Health, Division of Obstetrics and Gynaecology, Oslo University Hospital, Rikshospitalet, Norway; 3 Section of Specialised Endocrinology, Department of Medicine, Oslo University Hospital, Rikshospitalet, Norway; 4 Faculty of Clinical Medicine, University of Oslo, Rikshospitalet, Norway; 5 Division of Obstetrics and Gynaecology, Oslo University Hospital, Rikshospitalet, Norway

**Keywords:** Area under the curve, Curve shape, Functional data analysis, Functional principal component analysis, Gestational diabetes, Glucose curve, Glucose oscillations, Glucose variability, Oral glucose tolerance test, Pregnancy

## Abstract

**Background:**

Plasma glucose levels are important measures in medical care and research, and are often obtained from oral glucose tolerance tests (OGTT) with repeated measurements over 2–3 hours. It is common practice to use simple summary measures of OGTT curves. However, different OGTT curves can yield similar summary measures, and information of physiological or clinical interest may be lost. Our mean aim was to extract information inherent in the shape of OGTT glucose curves, compare it with the information from simple summary measures, and explore the clinical usefulness of such information.

**Methods:**

OGTTs with five glucose measurements over two hours were recorded for 974 healthy pregnant women in their first trimester. For each woman, the five measurements were transformed into smooth OGTT glucose curves by functional data analysis (FDA), a collection of statistical methods developed specifically to analyse curve data. The essential modes of temporal variation between OGTT glucose curves were extracted by functional principal component analysis. The resultant functional principal component (FPC) scores were compared with commonly used simple summary measures: fasting and two-hour (2-h) values, area under the curve (AUC) and simple shape index (2-h minus 90-min values, or 90-min minus 60-min values). Clinical usefulness of FDA was explored by regression analyses of glucose tolerance later in pregnancy.

**Results:**

Over 99% of the variation between individually fitted curves was expressed in the first three FPCs, interpreted physiologically as “general level” (FPC1), “time to peak” (FPC2) and “oscillations” (FPC3). FPC1 scores correlated strongly with AUC (*r*=0.999), but less with the other simple summary measures (−0.42≤*r*≤0.79). FPC2 scores gave shape information not captured by simple summary measures (−0.12≤*r*≤0.40). FPC2 scores, but not FPC1 nor the simple summary measures, discriminated between women who did and did not develop gestational diabetes later in pregnancy.

**Conclusions:**

FDA of OGTT glucose curves in early pregnancy extracted shape information that was not identified by commonly used simple summary measures. This information discriminated between women with and without gestational diabetes later in pregnancy.

## Background

Plasma glucose level is one of the most commonly used metabolic measures, both in research and in clinical settings [1-4]. In persons with normal glucose tolerance and metabolism, glucose levels rise after a dietary intake, and usually return to normal, postprandial levels after 2–3 hours [5,6]. For practical purposes, oral glucose tolerance test (OGTT) is used to define glucose tolerance [5,7,8]. Numerous studies have shown that high OGTT values are associated with an increased risk of adverse health outcomes [2-4,9], but there is no general agreement with respect to time points for glucose sampling during OGTT, cut-off values or test duration [1,2,4,10].

OGTT values are discrete, ordered measurements from an underlying, continuous process; i.e. an individual’s glucose regulation. Temporal OGTT measurements are often used to illustrate the underlying glucose curves, but the information inherent in the shape of these curves has been the subject of few studies [11-14]. It is common practice to use simple summary measures, such as fasting value, two-hour (2-h) value or area under the curve (AUC) to obtain information about an individual’s glucose tolerance. Simple summary measures are also frequently used in studies with continuous glucose monitoring [15,16]. To gain more information from OGTT glucose curves, simple shape summaries (shape indices), have been suggested [11-13]. However, different OGTT glucose curve trajectories can yield similar simple summary measures, and information of physiological or clinical interest may consequently be lost.

Functional data analysis (FDA) is a collection of statistical techniques specifically developed to analyse curve data [17-19]. When applying FDA, the entire curve is used as the basic unit of information, instead of the OGTT measurements at specific time points. FDA has been applied in some research disciplines during the last couple of decades, and has yielded novel insights of clinical importance in neuroscience [20], nephrology [21] and studies of gait [22,23]. An important FDA technique is functional principal component analysis (FPCA), which is used to extract the common temporal characteristics of a set of curves [18].

The main aim was to study the usefulness of FDA in the analysis of OGTT glucose curve trajectories. FDA, and in particular FPCA, was used to analyse OGTT data in a Norwegian prospective cohort study of healthy pregnant women [24]. We extracted temporal information from the shape of OGTT glucose curves and compared this to the information obtained from standard simple summary measures. By regression analyses we studied the OGTT glucose curves in relation to body mass index (BMI) categories in early pregnancy and gestational diabetes mellitus (GDM) later in pregnancy.

## Methods

### Participants and data

The STORK study is a prospective cohort of 1031 healthy pregnant women of Scandinavian heritage who registered for obstetric care at the Oslo University Hospital Rikshospitalet from 2001 to 2008 [25]. Exclusion criteria were multiple pregnancy, known history of type 1 or type 2 diabetes mellitus, and severe chronic diseases (pulmonary, cardiac, gastrointestinal, or renal). The overall aim of the STORK study was to gain insights into maternal metabolic syndrome and the determinants of foetal macrosomia [25]. Results of a 75 g OGTT, age, height and weight were recorded at inclusion at gestational weeks 14–16. Fifty-seven women (5.5%) with incomplete OGTT data were excluded, yielding a study sample of 974 women. During follow-up, 2-h glucose values at gestational weeks 30–32 were available for 930 (95%) women.

Venous blood samples were collected for OGTT in tubes containing Ethylenediaminetetraacetic acid (EDTA) between 07:30 and 08:30 after an overnight fast. Fasting glucose was measured immediately in a drop of fresh, whole EDTA blood, and further blood samples were taken every 30 minutes for 2 h, for a total of five OGTT measurements per woman. Glucose measurements were done by the Accu-Chek Sensor glucometer (Roche Diagnostics, Mannheim, Germany). Inter-assay coefficient of variation was <10%. Due to an unexpected increasing trend in fasting glucose values over the 7 years of participant recruitment, all glucose measurements were de-trended prior to the present analyses, as previously described in detail [26].

The study was approved by the Regional Committee for Medical Research Ethics, Southern Norway, Oslo, Norway (reference number S-01191), and performed according to the Declaration of Helsinki. All participating women provided written informed consent.

### Data description

Descriptive statistics were mean, standard deviation (SD) and range, or frequency and percentage. The study sample and women with incomplete OGTT data were compared by two-sample *t* tests or *χ*^2^ tests.

### Functional data analysis

FDA is a common term for statistical techniques specifically developed for analysing curve data [17-19]. In FDA a temporal set of observations is transformed into a single, functional object, and statistical analysis is then performed on this continuous function, rather than on the original discrete data points. This makes it possible to extract information from the temporal process as a whole, instead of merely point-by-point. In a sample of curves, the mean curve is used descriptively, as in traditional statistical analyses, and with proper modification, most standard statistical methods can be phrased in the framework of FDA. The principles of the analyses are explained hereafter, and technical details are given in the appendices.

### Curve fitting

The five OGTT measurements for the 974 participating woman were converted into 974 continuous, smooth curves by subject-specific spline smoothing with B-splines basis functions [17,19] (Appendix A). These individually fitted curves formed the basis for the subsequent FDA.

### Functional principal component analysis

FPCA was used to study the temporal variation in the 974 fitted curves. FPCA extracts a limited number of FPC curves that describe the temporal patterns associated with the largest proportions of the variation in the individual, fitted curves [17-19] (Appendix B). The FPC curves represent independent parts of the overall variability between the individual, fitted curves. The FPCA also yield individual FPC scores for each curve. The score variables are per definition independent, and the variation within the scores of an FPC quantifies the magnitude of the total variance explained by this FPC. A woman’s FPC score for an FPC curve reflects how her individual curve trajectory corresponds to the general temporal feature expressed by this FPC curve. By FPCA it is thus possible to study how OGTT glucose curve trajectories vary from woman to woman. FPC curves are often illustrated by plots showing how an individual curve differs from the mean curve if the FPC scores are high or low, rather than plots of the FPC curves directly [17-19]. As in traditional principal component analysis, FPCs may be interpreted and labelled according to the information they exhibit, which in turn can be related to more conventional physiological or clinical theories.

### Functional principal component scores vs simple summary measures

The Pearson correlation coefficient (*r*) was used to assess the associations between FPC scores, original glucose measurements and several simple summary measures of OGTT: fasting value, 2-h value, AUC and a simple shape index. We used the most cited simple shape index for OGTT [12], defined as the 2-h value minus the 90-min value for curves classified as “monophasic” or “biphasic”, and the 90-min value minus the 60-min value for curves classified as “triphasic”. The classification of curves, i.e. the determination of the number of phases within a curve involves an empirically chosen glucose threshold of 0.25 mmol/l [12]. Curves that did not meet the criteria for classification into mono-, bi- or triphasic were labelled “unclassified” and left out of the analyses.

### Functional analysis of variance

The relation between BMI and simple summary measures of glucose values is well-known [27]. Functional analysis of variance (FANOVA), the functional counterpart of traditional analysis of variance (ANOVA), was used to analyse the effect of BMI on the shape of OGTT glucose curves [18], using the fitted curves as responses. The WHO classification for BMI was utilised (underweight (<18.5 kg/m^2^), normal weight (18.5-25 kg/m^2^, reference category), overweight (25–30 kg/m^2^) and obese (≥30 kg/m^2^) [27]) and BMI was entered as a categorical explanatory variable. The analysis was based on the shape of the mean curve in each BMI category, and the temporal differences between these curves (Appendix C). In FANOVA, the effect estimates are themselves curves over the same time span as the curves under study, i.e. OGTT glucose curves. Functional 95% confidence intervals (CIs) and *p* curves were obtained for the difference between two mean curves. The FANOVA also gives an overall *p* value for the difference between two BMI categories.

### FANOVA vs ANOVA of simple summary measures

The simple summary measures described previously were compared across the BMI categories using traditional ANOVA, with Bonferroni corrected post hoc tests.

### Curve shape information in regression analyses

There is an on-going discussion about the diagnostic criterion for GDM [28,29]. However, as a new international consensus has yet to be established, we have kept the GDM definition which at present is recommended by the WHO: a 2-h OGTT value of 7.8 mmol/l or higher [1]. Consequently, the 2-h value is important in current clinical practice. The impact of the curve shape in early pregnancy on glucose intolerance later in pregnancy, i.e. the 2-h value at gestational weeks 30–32, was assessed by regression analyses, using the FPC scores at gestational weeks 14–16 as explanatory variables.

To visualise the clinical usefulness of the curve shape information more clearly, and to account for potential non-linear relations between variables, the 2-h values at gestational weeks 30–32 were grouped into seven categories and multinomial logistic regression was performed [30] using this categorised variable as the response. The categories were based on the diagnostic criterion for GDM and on assessments of group size and percentiles in the sample: <3.27 (2.5^th^ percentile), [3.27, 3.89) (2.5^th^-10^th^ percentile), [3.89, 6.39) (10^th^-75^th^ percentile; reference category), [6.39, 6.90) (75^th^-85^th^ percentile), [6.90, 7.8) (85^th^ percentile to diagnostic cut-off for GDM) [7.8, 8.84) (GDM diagnosis to 98^th^ percentile) and ≥8.84 mmol/l.

Five different models were fitted. Model 1 included BMI and the three independent FPC score variables from gestational weeks 14–16 as covariates, while models 2–5 included BMI and either the fasting value, the 2-h value, the AUC or the shape index, all from gestational weeks 14–16, as covariates. These simple measures were included one at a time in models 2–5, due to colinearity. Other covariates were not included in the models. It is beyond the scope of the article to build an extensive prediction model or to adjust for variables possibly on the causal pathway to the outcome. All covariates were continuous.

### Software

FDA, i.e. curve fitting, FPCA and FANOVA, were performed using the fda package in R 2.13.0 [31]. The multinomial regression was done by the mlogit package in R 2.13.0 [31]. The R script is available as supplementary material [see Additional file 1. All other analyses were performed in SPSS 19.

## Results

### Data description

Characteristics of the study sample at gestational weeks 14–16 are shown in Table 1. The women in the study sample were not significantly different from those with incomplete OGTT data (0.11≤*p*≤0.94). The number of women with a GDM diagnosis increased from 3 (0.3%) at gestational weeks 14–16 to 51 (5.5%) at gestational weeks 30–32 (Table 1).

**Table 1:** Sample characteristics

**Characteristic**	**Study sample, *n*=974**^**a**^		**Excluded**^**b**^**, *n*=57**^**a**^	**Total cohort, *n*=1031**^**a**^
		**Range**		
Gestational weeks	15.8 (1.3)	12.1-22.0	16.0 (1.4)	15.8 (1.3)
Age	31 (4)	19-42	31 (4)	31 (4)
Para 0	517 (54%)		28 (50%)	545 (53%)
Daily smoker^c^	27 (3%)		1 (2%)	28 (3%)
Height (cm)	169 (6)	150-184	169 (6)	169 (6)
Weight (kg)	69.9 (12.0)	44.6-123.1	68.2 (12.5)	69.8 (12.0)
BMI (kg/m^2^)	24.5 (3.9)	17.2-44.0	23.4 (3.8)	24.5 (3.9)
Birth weight^d^ (g)	3588 (570)	600-5420	3554 (671)	3586 (576)
Blood glucose (mmol/l), first trimester				
Fasting	4.0 (0.4)	2.6-5.3		4.0 (0.4)
30 min	5.7 (1.2)	2.5-9.7		5.7 (1.2)
60 min	5.0 (1.4)	2.0-10.9		4.9 (1.4)
90 min	4.5 (1.2)	2.0-10.1		4.5 (1.2)
2 h	4.1 (1.1)	1.2-7.8		4.1 (1.1)
GDM^e^: 2-h value≥7.8 mmol/l	3 (0.3%)			3 (0.3%)
Blood glucose (mmol/l), third trimester				
2 h	5.5 (1.3)	1.9-10.3		5.5 (1.3)
GDM^e^: 2-h value≥7.8 mmol/l	51 (5.5%)			54 (5.5%)

Data are mean (SD) or frequency (%).

^a^ Numbers may not add up to total due to missing data for some variables.

^b^ Women excluded due to incomplete OGTT data.

^c^ ≥1 cigarette/day.

^d^ Birth weight of offspring.

^e^ Gestational diabetes mellitus.

### Curve fitting

**Figure 1:**
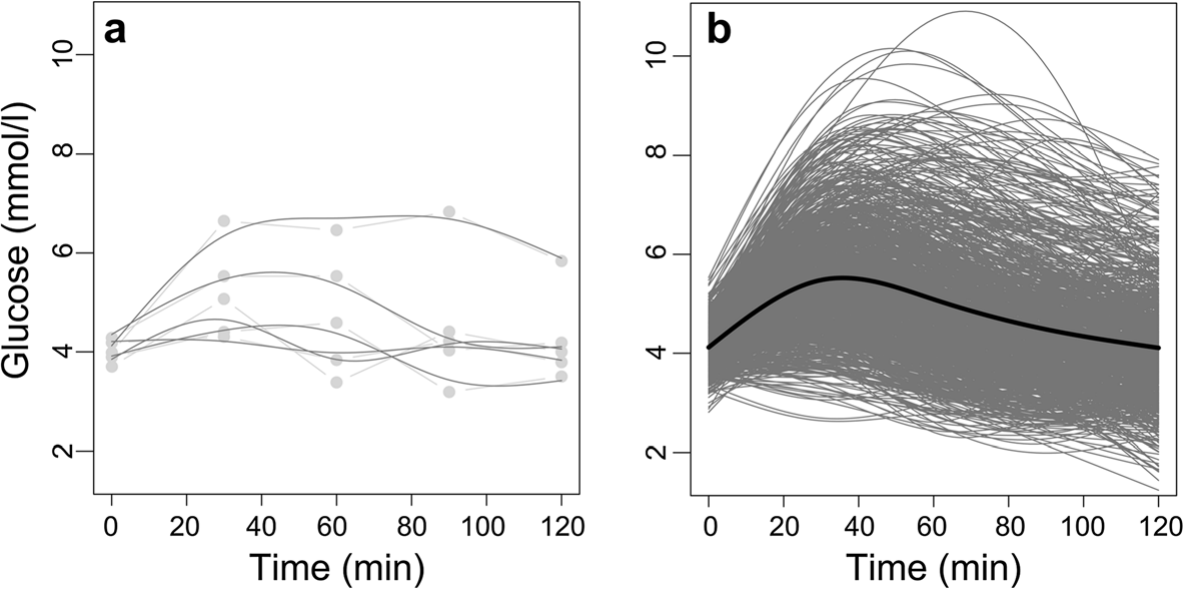
**Observed OGTT data and individually fitted curves at gestational weeks 14–16. a** shows the observed OGTT data (light grey) and individually fitted curves (dark grey) for the first five women in the study. The straight lines indicate measurements from the same woman. **b** shows the 974 individually fitted curves (grey) and the mean of these curves (black).

The individually fitted, smooth OGTT glucose curves at gestational weeks 14–16 showed large variations between the individual curves (Figure 1).

### Functional principal component analysis

**Figure 2:**
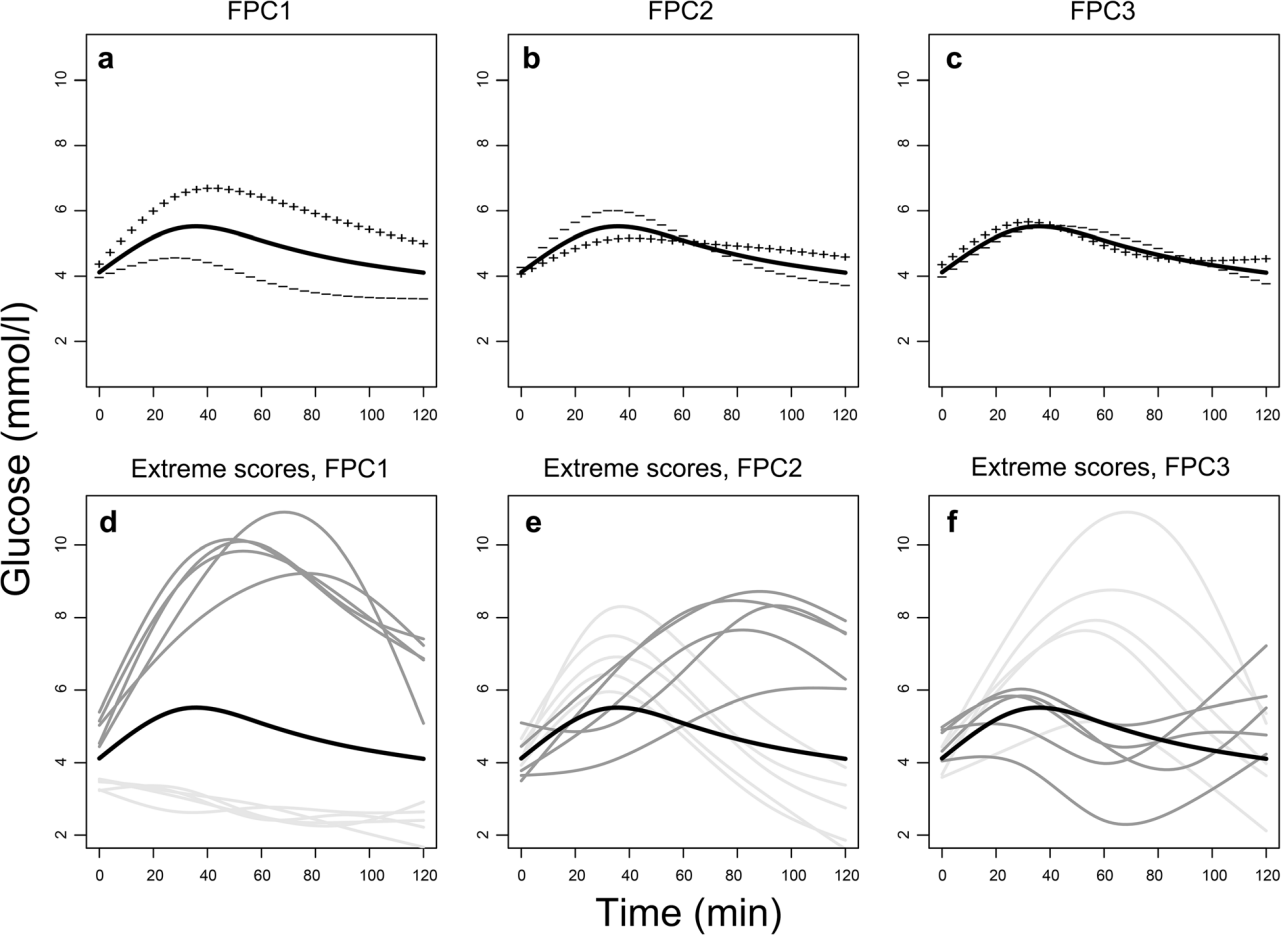
**Results from the FPCA. a-c** shows the mean of the fitted curves (solid line) and how the shape of an individual curve differs from the mean curve if a multiplum of the principal component curve (not shown) is added to (+ +) or subtracted from (− −) the mean curve. The multiplums correspond to one SD of the FPC1, FPC2 and FPC3 scores, respectively. **d-f** shows the mean of the fitted curves (black), and the individual curves for the five women with the highest positive scores (dark grey) and the five with the lowest negative scores (light grey) for each of the three FPCs.

The essential modes of temporal variation between the fitted curves were extracted by FPCA (Figure 2). The first FPC (FPC1, Figure 2a) explained 88.1% of the variation between the fitted curves, the second FPC (FPC2, Figure 2b) 8.6% and the third FPC (FPC3, Figure 2c) 2.4%, respectively. The corresponding physiological interpretations were the general glucose level (FPC1, “general level”), the time to peak for glucose (FPC2, “time to peak”) and the oscillations in OGTT glucose curves (FPC3 “oscillations”), respectively. Women with high FPC1 scores had generally high glucose levels compared with the mean glucose level (Figure 2a). Women with high FPC2 scores had a longer than average time to peak, and it took longer for their glucose levels to return to normal postprandial levels (Figure 2b). Women with high FPC3 scores had curves that oscillated faster than the mean (Figure 2c). The plots of the five women with the highest and lowest scores for each of the FPCs (Figure 2d-f) highlighted these physiological interpretations. In sum, more than 99% of the total variation between the individual curves was explained by the first three FPCs, and further analyses were therefore restricted to these three FPCs.

**Figure 3:**
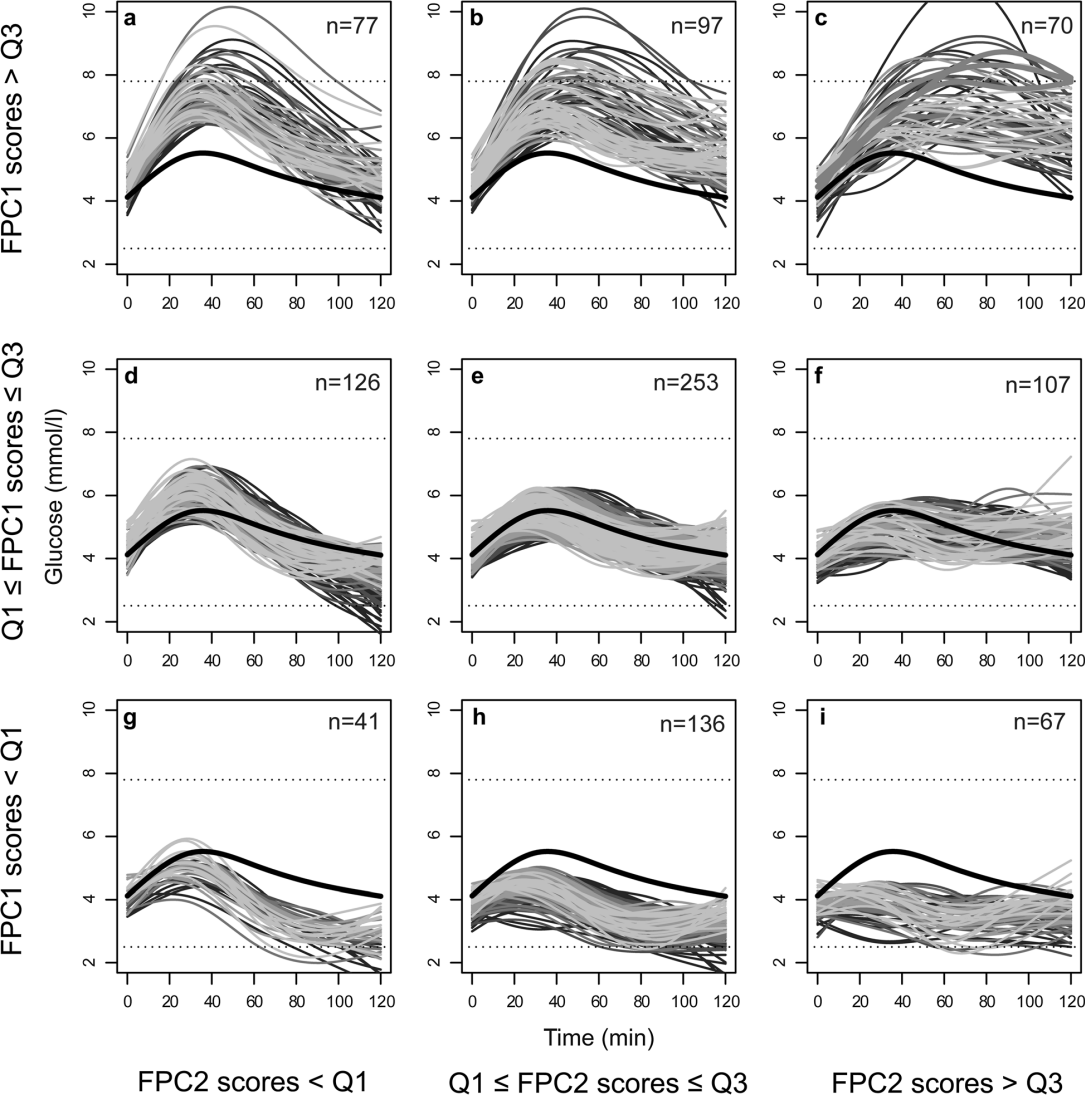
**Individual curves.** The figure shows the 974 individual, fitted curves classified according to the lower (Q1) and upper (Q3) quartiles of the FPC1 and FPC2 scores. The bold black curve is the overall mean of the fitted curves. Higher panels indicate higher FPC1 scores, and panels to the right represent higher FPC2 scores. The magnitudes of the FPC3 scores are represented using shades of grey: the lighter shades indicate higher FPC3 scores. The lower dashed line is 2.5 mmol/l, one possible cut-off for hypoglycaemia [32], and the upper dashed line is the diagnostic threshold for gestational diabetes, i.e. a 2-h value of 7.8 mmol/l [1]. The three women diagnosed with gestational diabetes are outlined with bold, grey lines in Figure 3c.

For the majority of the women (89%), the entire OGTT glucose curve was between 2.5 and 7.8 mmol/l, while 6% had hypoglycaemic levels (values <2.5 mmol/l [32]) and three women were diagnosed with GDM. The 974 individual, fitted curves are grouped according to the lower and upper quartiles of the FPC1 and FPC2 scores in Figure 3. Women with high scores for both FPC1 and FPC2 had the highest glucose levels (Figure 3c), and these included the three women with GDM. Several women had OGTT glucose curve trajectories similar to those of the three GDM cases, but their curves descended below the GDM diagnosis threshold just before 2 h (Figure 3c).

### Functional principal component scores vs simple summary measures

The FPCA transformed the five correlated OGTT measurements (0.40≤*r*≤0.84) into three uncorrelated FPC scores reflecting three distinct temporal features (Table 2). In contrast to fasting value, the 2-h value was positively associated with all three FPC scores (0.37≤*r*≤0.79). AUC was highly correlated with the FPC1 scores (*r*=0.999) but not with the FPC2 and FPC3 scores (*r*=−0.01 and *r*=0.05, respectively). The shape index was calculated as the 2-h value minus the 90-min value for 587 (60%) women, and as the 90-min value minus the 60-min value for 124 (13%) women. A total of 263 (27%) curves failed to meet the classification criteria of the shape index and were left out of these analyses. The shape index was most strongly associated with the FPC3 score (*r*=0.67). Pairwise scatter plots of these bivariate associations (not shown) showed that the three women classified as having GDM did not exhibit unusual FPC scores. Their FPC1 and FPC2 scores were high, but 33 other women had FPC1 scores in the same range, and 12 of them also had FPC2 scores above the upper quartile.

**Table 2:** **Pearson correlation coefficients for OGTT measurements, FPC scores and simple summary measures (*n*=974)**

**OGTT**	**OGTT**					**FPC scores**		
	**Fasting**	**30 min**	**60 min**	**90 min**	**2 h**	**FPC1: “General level”**	**FPC2: “Time to peak”**	**FPC3: “Oscillation”**
Fasting	1.00	0.44	0.40	0.41	0.42	0.47	−0.12	0.42
30 min		1.00	0.77	0.66	0.55	0.85	−0.47	0.19
60 min			1.00	0.84	0.70	0.96	−0.04	−0.22
90 min				1.00	0.80	0.93	0.31	−0.01
2 h					1.00	0.79	0.40	0.37
AUC	0.50	0.86	0.95	0.92	0.81	0.999	−0.01	0.05
Simple shape index^a^	−0.10	−0.34	−0.49	−0.41	0.12	−0.42	0.21	0.67

^a^*n*=711. Calculated as 2-h value minus 90-min value, or 90-min minus 60-min value [12].

### Functional analysis of variance

**Figure 4:**
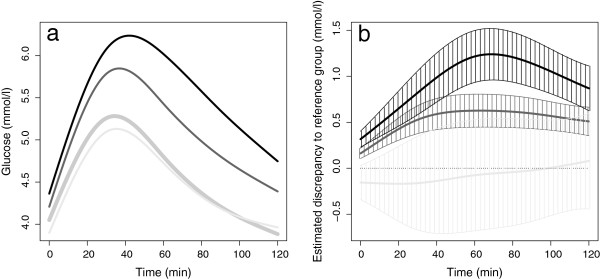
**Results of the FANOVA. a** shows the means of the fitted glucose curves for the BMI categories underweight (n=17, light grey curve), normal weight (n=588, bold grey curve), overweight (n=274, dark grey curve) and obese (n=87, black curve). **b** shows the estimated functional regression coefficients with corresponding CIs (shaded) and with normal weight as the reference category.

**Figure 5:**
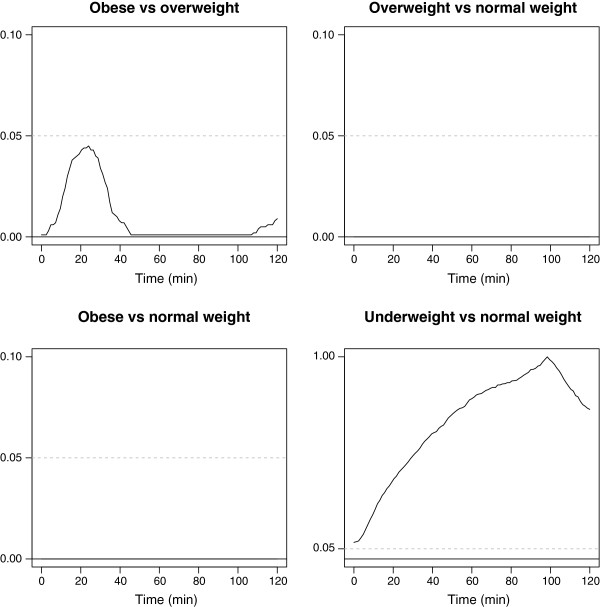
***p*curves for pairwise comparisons of BMI categories using functional permutation tests.** The dashed line is the significance level of 0.05.

The means of the fitted curves differed between the four BMI categories (Figure 4a). While the curvature was similar, there were clear vertical shifts between the mean curves for normal weight, overweight and obese women. The functional CIs for the differences between underweight, overweight and obese women, as compared to normal weight women, are shown in Figure 4b. Pairwise comparisons of BMI categories showed the time periods of OGTT where the mean curves differed, as illustrated by the *p* curves in Figure 5. We found overall statistically significant differences between obese and overweight women (*p*<0.001), obese and normal weight women (*p*<0.001) and overweight and normal weight women (*p*<0.001). No statistically significant difference was found between underweight and normal weight women (*p*=0.26).

### FANOVA vs ANOVA of simple summary measures

The results from ordinary ANOVA comparing the BMI categories in regard to fasting value, 2-h value or AUC were similar to those of the FANOVA comparisons. However, the shape index was only significantly different between obese and normal weight women (data not shown).

### Multinomial regression with FPC scores

**Figure 6:**
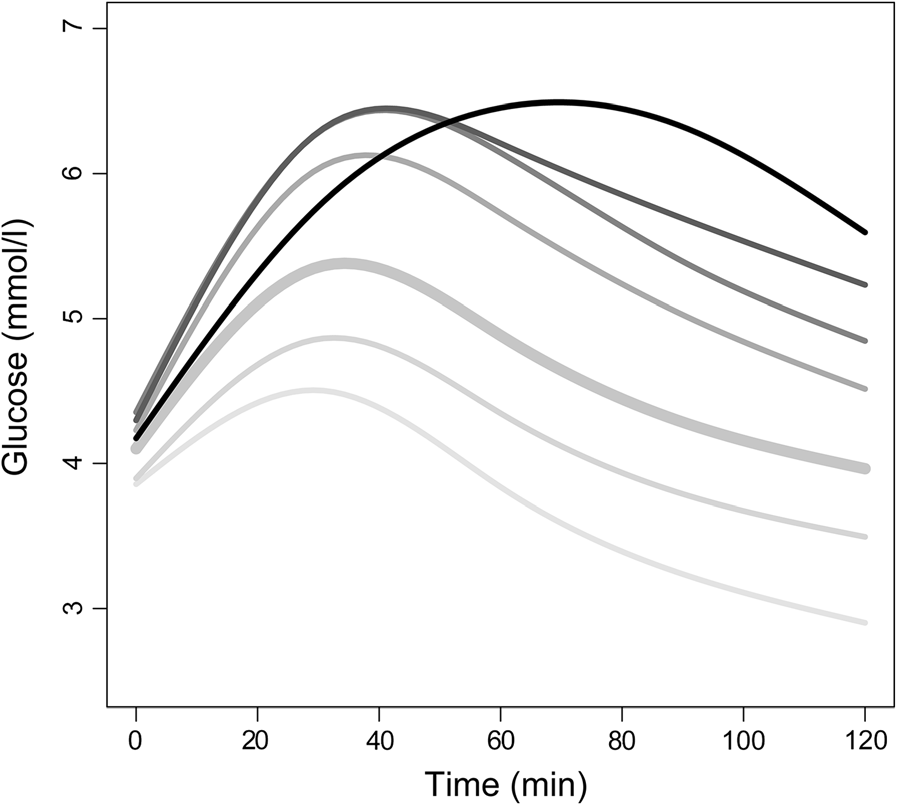
**Means of glucose curves in first trimester, for different glucose categories later in pregnancy.** The figure shows the means of the fitted glucose curves at gestational weeks 14–16, for different categories of 2-h values at gestational weeks 30–32. Darker lines indicate higher 2-h values. The 2-h glucose categories are <3.27, [3.27, 3.89), [3.89, 6.39), [6.39, 6.90), [6.90, 7.8), [7.8, 8.84) and ≥8.84 mmol/l.

The means of the fitted curves at gestational weeks 14–16 for the seven pre-defined categories of 2-h values at gestational weeks 30–32 are shown in Figure 6. The women in the two upper categories (*n*=51) were all diagnosed with GDM at gestational weeks 30–32, but the mean curves in these two subgroups displayed different pathophysiology at gestational weeks 14–16. All women in the five lowest categories had a 2-h value below 7.8 mmol/l at gestational weeks 30–32, and were thus not diagnosed with GDM, but there were clear vertical shifts between their mean OGTT glucose curves at gestational weeks 14–16.

The results of the multinomial logistic regression analyses are shown in Table 3. The FPC1 scores and the AUC (Models 1 and 4, respectively) yielded nearly identical results, thus the results for AUC are not shown. We found that the mean FPC1 scores (and AUC) in the reference category were significantly different from the mean FPC1 scores in all other categories (all *p*<0.001), but that the mean FPC1 scores in subgroups of women with GDM were not significantly different (*p*=0.40). Also, the mean FPC1 scores in the lowest GDM category were not significantly different from the mean FPC1 scores in the closest non-GDM category (*p*=0.59). Similarly, no significant differences were found for fasting value, 2-h value or shape index in the three upper categories, i.e. between subgroups of women with and without GDM. In contrast, FPC2 scores discriminated between women who did and did not develop GDM, and between subgroups of women diagnosed with GDM later in pregnancy. The means of the FPC2 scores were significantly different between the three upper categories, *p*=0.01 and *p*=0.02, respectively. We also found a difference in the FPC3 scores between the two GDM categories (*p*=0.05) (Table 3).

**Table 3:** Results from four multinomial logistic regression analyses

		Model 1: FPC1, FPC2 and FPC3 scores, gestational weeks 14–16*											
**2-h value, gestational weeks 30-32**	***n***	**FPC1 scores**^**a**^				**FPC2 scores**				**FPC3 scores**			
		**Mean (SD)**	**OR (95% CI)**	***p***	***p***^**b**^	**Mean (SD)**	**OR (95% CI)**	***p***	***p***^**b**^	**Mean (SD)**	**OR (95% CI)**	***p***	***p***^**b**^
≥8.84	19	12.6 (13.5)	1.08 (1.04,1.13)	<0.001	0.40	5.7 (5.1)	1.36 (1.20,1.53)	<0.001	0.01	−0.7 (3.0)	0.87 (0.68,1.10)	0.23	0.05
[7.8,8.84)	32	11.1 (13.1)	1.11 (1.07,1.14)	<0.001	0.59	1.8 (4.7)	1.14 (1.04,1.25)	0.01	0.02	0.5 (1.6)	1.14 (0.95,1.37)	0.16	0.41
[6.90,7.8)	83	9.4 (12.8)	1.10 (1.07,1.12)	<0.001	0.02	−0.1 (3.1)	1.01 (0.95,1.08)	0.69	0.60	0.2 (1.9)	1.05 (0.93,1.19)	0.47	0.57
[6.39,6.90)	94	4.9 (9.6)	1.06 (1.04,1.09)	<0.001		−0.5 (3.8)	0.99 (0.93,1.06)	0.79		0.1 (1.8)	1.00 (0.89,1.13)	0.98	
[3.89,6.39)	601	−1.8 (9.7)	1	Ref		−0.2 (3.4)	1	Ref		0.1 (1.8)	1	Ref	
[3.27,3.89)	70	−6.9 (8.7)	0.94 (0.91,0.98)	<0.001	<0.01	0.0 (3.0)	0.98 (0.90,1.06)	0.62	0.07	−0.5 (1.7)	0.85 (0.74,0.98)	0.03	0.63
<3.27	23	−12.0 (9.8)	0.83 (0.78,0.90)	<0.001		−0.9 (3.8)	0.85 (0.73,0.98)	0.02		−0.8 (2.4)	0.80 (0.62,1.01)	0.07	
		Model 2: Fasting value gestational weeks 14-16*				Model 3: 2-h value gestational weeks 14-16*				**Model 5: Simple shape index**^**c**^gestational weeks 14-16*			
		**Mean (SD)**	**OR (95% CI)**	***p***	***p***^**b**^	**Mean (SD)**	**OR (95% CI)**	***p***	***p***^**b**^	**Mean (SD)**	**OR (95% CI)**	***p***	***p***^**b**^
≥8.84	19	4.1 (0.5)	2.00 (0.57,6.86)	0.28	0.55	5.5 (1.4)	3.40 (2.24,5.18)	<0.001	0.71	−0.80 (1.3)	0.53 (0.30,0.92)	0.03	0.36
[7.8,8.84)	32	4.1 (0.3)	3.17 (1.22,8.01)	0.02	0.54	5.3 (1.4)	3.11 (2.24,4.33)	<0.001	0.07	−0.47 (0.8)	0.73 (0.46,1.17)	0.19	0.92
[6.90,7.8)	83	4.2 (0.4)	4.43 (2.26,7.71)	<0.001	0.03	4.9 (1.1)	2.25 (1.79,2.84)	<0.001	0.01	−0.47 (0.8)	0.71 (0.52,0.97)	0.03	0.84
[6.39,6.90)	94	4.1 (0.4)	1.87 (0.99,3.25)	0.04		4.4 (0.9)	1.58 (1.26,1.98)	<0.001		−0.51 (0.7)	0.74 (0.55,1.00)	0.05	
[3.89,6.39)	601	4.0 (0.4)	1	Ref		4.0 (0.9)	1	Ref		−0.29 (0.7)	1	Ref	
[3.27,3.89)	70	3.8 (0.3)	0.32 (0.15,0.65)	<0.01	0.63	3.5 (0.8)	0.57 (0.42,0.78)	<0.001	<0.01	−0.28 (0.7)	0.95 (0.66,1.37)	0.80	0.18
<3.27	23	3.8 (0.4)	0.23 (0.09,0.93)	0.01		2.9 (0.4)	0.24 (0.14,0.41)	<0.001		−0.33 (1.1)	0.60 (0.34,1.07)	0.09	

^a^ The FPC1 scores in model 1 and the AUC in model 4 yielded nearly identical results and the AUC results are thus not shown.

^b^*p* values from pairwise comparison between adjacent groups.

^c^*n*=711. Calculated as 2-h value minus 90-min value, or 90-min minus 60-min value [12].

* Categories of 2-h values in the third trimester is the response variable and OGTT characteristics in gestational weeks 14–16 are explanatory variables. All models are adjusted for BMI in gestational weeks 14–16.

## Discussion

The present study demonstrated how information inherent in the shape of OGTT glucose curves can be extracted. The FDA approach yielded quantifiable shape entities with physiologically interpretable information that was not contained in the traditional simple summary measures. The extracted shape information differed significantly between women who did and did not develop GDM, and between subgroups of women diagnosed with GDM later in pregnancy, while various simple summary measures did not.

The challenge of extracting shape information from glucose curves has been addressed by others [11-14], but these studies have focused on either simple shape indices or advanced parametric modelling. The present study is the first to use statistical tools and corresponding available software developed specifically for curves, to analyse OGTT data.

Our results were based on a large and relatively homogenous sample of healthy, pregnant women, but on a small number of glucose measurements per woman, as compared to those of an intravenous glucose tolerance test. One might expect to find even more physiologically interesting details and discriminating features of OGTT glucose curves, e.g. a larger number of FPCs with a substantial percentage of explained variability and more temporal details in the FPCs, in a more heterogeneous population with a more frequent OGTT sampling. For instance, our fitted curves could not reveal more than two peaks, but curves based on more densely sampled measurements over a longer time period than 2 h would likely show decreasingly oscillating curves rather than purely biphasic trajectories [14]. We therefore proposed the term “oscillating” as a qualitative description of OGTT glucose curves with more than one peak rather than using the term “biphasic”, which has been used by others [12,14]. Furthermore, the classification of OGTT glucose curves as “biphasic”, “monophasic” or “unclassified”, involves several ad hoc conditions [12]. In the present study, we used FPC scores as continuous variables, as per general statistical recommendations, as this is the first choice of analysis in order to retain information and statistical power [33].

The mean of the fitted curves obtained from FDA (Figures 1, 2, 3) corresponded well with the familiar general shape of OGTT glucose curves [6,34,35]. In the literature in general, figures and analyses are usually based on the means at selected time points, with variability quantified by the SD or SE at the same time points, e.g. when comparing glucose responses [6]. In general, as seen in Figures 1, 2 and 3, the temporal mean under-communicates the temporal variability. Although individual glucose curves have been presented in several publications [14,35,36], the variability in curve trajectories is highly under-reported, and thus largely unknown. As a result, the information indicated by the shape of OGTT glucose curves is rarely used in clinical practice, and only occasionally in research, although the standard practice of taking repeated blood samples during OGTT suggests a focus on the curve. We have presented the individual, fitted curves in order to emphasise the heterogeneity between our study women and to provide a reference for OGTT glucose curves in healthy, pregnant women.

While a FPCA will decompose the variation between individual curves into a set of uncorrelated, temporal features, the clinical usefulness of this analysis depends on how the FPCs are interpreted. In this study, current insight into metabolism supported the interpretations of the FPCs as plausible and important physiological features. FPC1, which represented the general level and was the most important temporal feature of the curves, was almost perfectly correlated with AUC, and was significantly higher in women with high BMI. The fasting value and the 2-h value were also correlated with FPC1, but not as strongly as AUC. This is to be expected as a single measurement from a temporal phenomenon rarely describes the most essential temporal feature of the corresponding curve satisfactorily. Moreover, AUC is much better than the widely used fasting, or 2-h value in capturing the essential temporal information of OGTT glucose curves, which is consistent with results from previous studies [37-39]. The strongest association between the shape index and the FPC scores was found for FPC3 scores, which explained the smallest proportion of the total variance. This proportion was so small that FPC3 could have been left out of the analyses. We chose to include FPC3 for the comparison of FDA with the shape index. The shape index is based on an a priori classification of curves, involving an ad hoc set threshold for change. Many curves (27%) failed to meet the classification criteria and were left out of the analyses, resulting in a severe reduction of power and a biased representation of metabolic profiles in the study sample. Another, recently suggested shape index [13] is based on a rough approximation of the mean of the second order derivatives in the intervals between the measurements during the OGTT, giving a rough approximation of the total curvature. In the present study, FPC3 scores, representing the smallest proportion of the variance, quantified the amount of curvature. The shape feature of FPC3 was however less clear than for the first two components, and although it is possible that the third component might explain a larger part of the total variation if the sampling was more frequent and over a longer time period, this component should be used and interpreted with caution.

Glucose tolerance early in pregnancy has been found to predict glucose tolerance later in pregnancy [40]. The FPC1 scores, 2-h values and AUC differed significantly between groups of women without a GDM diagnosis at gestational weeks 30–32. However, only FPC2 scores were significantly different between women with and without GDM and only FPC2 and FPC3 scores differed significantly between diabetic women with the highest and second highest 2-h values in the third trimester. Thus, FPC1 or AUC alone did not capture all of the essential information about the differences in glucose metabolism. To distinguish curve trajectories reflecting deviating glucose tolerance from those considered normal, the information from FPC2 and FPC3 was necessary. A study of type 1 diabetes mellitus patients with islet transplantations showed that increased glucose AUC and time to peak C-peptide after metabolic testing were metabolic markers of islet allograft dysfunction [41], supporting the physiological importance of both FPC1 and FPC2 scores. The timing of the peak C-peptide was also found to be predictive of progression to type 1 diabetes mellitus in the Diabetes Prevention Trial [42].

The alternative to data-driven approaches such as FPCA for analysing full glucose curves is parametric modelling based on differential equation models of physiological mechanisms. Current concepts of blood glucose dynamics have been summarised in such models [14,43-45]. For instance, blood glucose levels and, hence, the shapes of glucose curves are affected by a number of key organs and physiologic processes that regulate the entry and removal of glucose from the blood [12,46]. A major disadvantage of parametric models is that estimating each person’s individual parameters requires many measurements, often based on intravenous test procedures [47]. Although the use of OGTTs is debated [48], it is the simplest and most frequently used test procedure in larger studies because “gold-standard” intravenous procedures such as the euglycaemic clamp [49] are time-consuming, invasive and labour intensive.

Another important issue with parametric models of blood glucose regulation is the “closed loop” assumption, which can be hard to justify when modelling biological processes in the body because such processes are usually also susceptible to external influences. Diet, physical activity, obesity, changes in weight or visceral fat deposits, smoking and stress have all been shown to affect blood glucose levels [35] and external factors can have long-term effects on metabolism [50]. The genetic disposition of each individual adds to this complexity [51]. Finally, pregnancy causes alterations in a wide range of variables, including hormonal changes, insulin resistance and alterations in daily life habits. Nevertheless, parametric models seldom adjust for confounding by external variables [14,44,45]. Hence, even when parametric models seem to fit the data well, the error term for fit can include structural information not addressed in the pre-defined model, including information on the long-term effects of diet and the endocrine changes caused by pregnancy itself. This can make it difficult to validate the physiological theories underlying parametric models.

Although FDA or parametric modelling are the most natural approaches to glucose data for the study of glucose curves as single entities, there are alternatives to these analyses for the data presented in this article. For instance, the relation between BMI and glucose values could have been examined with a classical longitudinal data analysis with five repeated measurements per woman, with random effect of woman and modelling of the covariance structure. Also, instead of scores from FPCA, ordinary PCA scores based on the five glucose variables could be used as input to the regression analysis of glucose tolerance later in pregnancy. With only five measurements per curve, and measurements taken at the same time points for each woman, such traditional multivariate methods would be expected to extract similar information as the FDA. However, FDA is easier to apply in situations with more frequent sampling, sampling at unequal time points and missing data. In addition, FDA emphasizes the basic assumption about continuity of the underlying process and its derivatives, and opens for analysis of the derivatives of the curves.

Contrary to general statistical advice [33], we have categorised two continuous variables in the analyses. An important aim of the present work was to introduce FDA and its benefits to a clinical audience. To ease the presentation of FDA, we chose to categorise BMI and the 2-h glucose at gestational weeks 30–32, based on the use of these variables in clinical practice. Different BMI categories are assumed to represent different risk groups [27], and BMI categories are frequently reported in clinical literature. The categorised BMI variable was therefore used in the analyses, although functional regression with BMI as a continuous variable would be preferable from a statistical point of view [33], especially as there were no obvious signs of nonlinearity (Figure 4a). The categorisation of the 2-h glucose value at gestational weeks 30–32, in contrast, revealed important non-linear relations (Figure 6). As an alternative to the multinomial logistic regression model, a regression model with the 2-h value as a continuous response variable could have been used.

The women in the cohort underwent two OGTTs, but only one was considered functional in the present work. We chose the 2-h value in third trimester as the main outcome instead of the entire curve in third trimester, due to the clinical relevance of this value in pregnancy care. As glucose curves are not commonly used, inference about the 2-h value would better illustrate the usefulness of information from FDA for a maternal pregnancy outcome in clinical practice.

Continuous glucose monitoring devices allow for more frequent glucose sampling over longer periods and might increasingly be used in future studies and in individual patient care to obtain OGTT measurements and measurements of glucose profiles in daily life. An increasing use of continuous glucose monitoring advocates the use of statistical tools that can properly analyse the continuous stream of data by providing curves that may be subjected to FDA as illustrated in the current work.

Furthermore, comparison of curve shape information from individuals with insulin resistance or beta cell failure might reveal whether curve features can distinguish between these two main processes that lead to the development of diabetes. Also, the curve shape information as obtained by FPCA in early pregnancy has the potential to predict complications in later pregnancy better than simple summary measures.

Our work shows that the FDA approach worked well, despite the very limited number of measurements for each participant. Dynamic, physiological processes will often be represented by scarcely sampled measurements, especially when repeated blood samples are required. In addition to glucose regulation, other examples where an FDA approach can be valuable include diurnal measurements of hormone regulation, metabolic changes during or after meals, or after physical exercise. The presented techniques should therefore also be explored in studies of metabolic disorders in non-pregnant populations.

## Conclusions

In conclusion, the FDA approach was superior to traditional analyses of OGTT data in terms of providing physiologically interpretable and important temporal information, and in terms of differentiating between women who did and did not develop GDM during pregnancy. We recommend the FDA approach for the analysis of glucose data sampled repeatedly during glucose tolerance testing, or continuous glucose monitoring, to capitalise on important information that would otherwise be lost.

## Appendix A

A.1. Curve fitting in functional data analysis

Let *y*_*i*_(*t*_*j*_) be the measurement from individual *i* at time *t*_*j*_, *i* = 1, …, *n* and *j* = 1, …, *J*. In our OGTT data, *n* = 974 and *J* = 5. To each individual set of observations, *y*_*i*_(*t*_*j*_), *j* = 1, …, *J*, we fit a continuous, smooth function *x*_*i*_(*t*), spanning the observed time range. In our OGTT data, *t* ∈ [0, 120]. The estimation of the continuous curves *x*_*i*_(*t*) from data points *y*_*i*_(*t*_*j*_) is based on the measurement model

(1) $$y_{i}\left(t_{j}\right)=x_{i}\left(t_{j}\right)+\epsilon_{\mathrm{ij}},$$

where *x*_*i*_(*t*_*j*_) is *x*_*i*_ evaluated at time *t*_*j*_ and *ϵ*_*ij*_ ~ N(0, *σ*^2^) is an error term. It can be shown that a smooth curve is well approximated by a linear combination of a set of smooth basis functions *ϕ*_*k*_(*t*), *k* = 1, …, *K*,

(2) $$x_{i}\left(t\right)\approx \sum_{k=1}^{K}c_{\mathrm{ki}}\phi_{k}\left(t\right)=c_{i}{}^{T}\phi \left(t\right),$$

where *c*_*ki*_ is the coefficient for the *k*^th^ basis function, **c**_*i*_ = (*c*_1*i*_, …, *c*_*Ki*_), and *ϕ*(*t*) = (*ϕ*_1_(*t*), …, *ϕ*_*K*_(*t*)). We apply B-spline basis functions, placing a knot at each of the *J* time points. With *ϕ*_*k*_(*t*_*j*_) denoting the *k*^th^ basis function evaluated at time *t*_*j*_, substituting (2) into (1) yields

(3) $$\begin{array}{l} \left[\begin{array}{lll} y_{1}\left(t_{1}\right) & \cdots & y_{n}\left(t_{1}\right)\\ \vdots & & \vdots \\ y_{1}\left(t_{J}\right) & \cdots & y_{n}\left(t_{J}\right) \end{array}\right]=\left[\begin{array}{ccc} \phi_{1}\left(t_{1}\right) & \cdots & \phi_{K}\left(t_{1}\right)\\ \vdots & & \vdots \\ \phi_{1}\left(t_{J}\right) & \cdots & \phi_{K}\left(t_{J}\right) \end{array}\right]\;\left[\begin{array}{lll} c_{11} & \cdots & c_{1n}\\ \vdots & & \vdots \\ c_{K1} & \cdots & c_{\mathrm{Kn}} \end{array}\right]\\ \;+\left[\begin{array}{lll} \epsilon_{11} & \cdots & \epsilon_{1n}\\ \vdots & & \vdots \\ \epsilon_{K1} & \cdots & \epsilon_{\mathrm{Kn}} \end{array}\right]\;, \end{array}$$

which in matrix notation reads

Y=ΦC+E,

with **Y**, **Φ**, **C** and defined from (3). Here **Y** is the *J* × *n* matrix of observed blood glucose measurements; **Φ** is the *J* × *K* matrix of the values of the *K* basis functions evaluated at times *t*_*j*_, and the *J* × *n* matrix of error terms. Finally, **C** is the *K* × *n* matrix of unknown linear coefficients *c*_*ki*_, which we estimate by minimising the penalised least squares expression

$${\left(Y-\mathrm{\Phi C}\right)}^{T}\left(Y-\mathrm{\Phi C}\right)+\lambda C^{T}\mathrm{RC}.$$

The penalty term, *λ***C**^*T*^**RC**, where *λ* is a smoothing parameter that defines the degree of regularisation, is added to compensate for random error, and is based on the total curvature of the fitted curve,

$$R=\int D^{2}\phi \left(s\right)D^{2}\phi^{T}\left(s\right)\mathrm{ds},$$

where *D*^2^*ϕ*(*s*) is the second derivative of the vector of basis functions *ϕ*(*t*). The smoothing parameter *λ* ∈ [0, *∞*) is estimated by optimising a generalised cross-validation criterion. For more detail, see publications by Ramsay et al [17,18].

### Appendix B

B.1. Functional principal component analysis

Functional principal component analysis (FPCA) can be viewed as rotating functional data to optimal empirical continuous basis functions, referred to as functional principal component (FPC) curves [17,18]. Associated with each FPC curve are individual FPC scores. These quantify how much the individual, fitted curves differ from the mean curve, in terms of the temporal pattern described by each FPC curve. An FPC curve *ξ*_*κ*_(*t*) and its corresponding FPC scores *z*_*κi*_, *κ* = 1, …, , for individuals *i* = 1, …, *n*, are estimated simultaneously by finding a weight function *ξ*(*t*) defined over the same range of *t* as the functional data *x*_*i*_(*t*), maximising the variance of the corresponding individual FPC scores *z*_*i*_, given by *z*_*i*_ = ∫ *ξ*(*t*)*x*_*i*_(*t*)*dt*, subject to constraints. The first FPC, *ξ*_1_(*t*), is found by maximising the variance of the principal component scores *z*_1*i*_ subject to the constraint∫ *ξ*_1_(*t*)^2^*dt* = 1. Consecutive FPCs are defined similarly under the additional constraint of being orthogonal to the already extracted FPCs. For more detail, see publications by Ramsay et al [17,18].

#### Appendix C

C.1. Functional analysis of variance

Functional analysis of variance (FANOVA) is a method for studying the difference between the functional means of fitted curves in mutually exclusive subgroups of the study sample.Consider a categorisation of the study sample into *g* = 1, …, *G* categories, e.g. BMI categories. Let *L*_*g*_ be the sample size in category *g*. We model the *l*^th^ OGTT glucose curve, *l* = 1, …, *L*_*g*_ in the *g*^th^ category, *x*_*lg*_(*t*), as

$$x_{\mathrm{lg}}\left(t\right)=\beta_{\mathrm{ref}}\left(t\right)+\beta_{g}\left(t\right)+\epsilon_{\mathrm{lg}}\left(t\right).$$

Here *β*_ref_(*t*) is the mean of the fitted curves in the reference category, *β*_*g*_(*t*) the difference between the mean curve in the *g*^th^ category and the reference category, and *ϵ*_*ig*_(*t*) the individual residual curve. The estimated group mean curve differences ${\hat{\beta }}_{g}\left(t\right),g=1,\ldots ,G,$ called the FANOVA coefficients, are based on the fitted curves described in Appendix A. They are also functions over the same *t* range.Differences between categories can be evaluated by functional CIs for the FANOVA coefficients, corresponding *p*(*t*) curves and overall *p* values from permutation *F* tests. The presented permutation tests are based on 1000 permutations of the fitted curves in two different categories. The CIs and *p*(*t*) curves are calculated point-wise over the *t* range, using the estimated F-ratio $\mathrm{FR}\left(t\right)=\frac{\mathrm{MRS}\left(t\right)}{\mathrm{MSE}\left(t\right)},$ calculated as the ratio of residual variance, *MRS*(*t*), to predicted variance, *MSE*(*t*). The permutation distribution is found for the point-wise F-statistic, giving CIs and *p*(*t*) curves over the *t* range, and for the maximal value of the point-wise F-statistic, giving an overall *p* value. For more detail, see publications by Ramsay et al [17,18].

## Abbreviations

2-h: Two-hour; ANOVA: Analysis of variance; AUC: Area under the curve; BMI: Body mass index (kg/m^2^); CI: Confidence interval; EDTA: Ethylenediaminetetraacetic acid; FANOVA: Functional analysis of variance; FDA: Functional data analysis; FPC: Functional principal component; FPCA: Functional principal component analysis; GDM: Gestational diabetes mellitus; OGTT: Oral glucose tolerance test; SD: Standard deviation.

## Competing interests

The authors declare that they have no competing interests.

## Authors’ contribution

TH, JB, NV and KG contributed to the design of the STORK study and acquisition of data. KFF, JR and MBV designed the data analysis and KFF analysed the data. All authors contributed to the statistical or clinical interpretation of the results, writing and revising the manuscript, and approved the final version.

## Pre-publication history

The pre-publication history for this paper can be accessed here:

http://www.biomedcentral.com/1471-2288/13/6/prepub

## Supplementary Material

Additional file 1 R script for functional data analysis of glucose curves. Click here for file
