# Supplementary material for: Shape information from glucose curves: Functional data analysis compared with traditional summary measures
Source: BMC Med Res Methodol. 2013 Jan 17;13:6. doi: 10.1186/1471-2288-13-6 (PMC3570313; doi:10.1186/1471-2288-13-6)
Supplement: Additional file 1 — R script for functional data analysis of glucose curves. [file 1471-2288-13-6-S1.pdf]

```
# R script for the functional data analysis (FDA) of early pregnancy OGTT data, as presented in the article
# Functional data analysis extracted physiologically important information from the shape of oral glucose tolerance test curves
# in a prospective cohort of pregnant women
# by KF Frøslie et al.

        # NB You might have to install the following R packages:
        # install.packages("foreign")
        # install.packages("fda")
        # install.packages("car")
        # install.packages("mlogit")
        # install.packages("Epi")


        # Read SPSS data, sort data and remove missing

library(foreign)
spssdata        <- as.data.frame(read.spss("M:/ArticleFDA/STORKdatabase.sav"))
spssdata        <- spssdata[order(spssdata$id),]
g               <- na.omit(cbind(spssdata$g0m,spssdata$g30m,spssdata$g60m,spssdata$g90m,spssdata$g2h))   # dim: 974,5

breaksuse       <- c(0,30,60,90,120)
ng              <- length(g[,1])

        # Plot of raw data

matplot(breaksuse,t(g[1:ng,]), type="l", lty=1, col="dark grey",lw=1,xlab="Time in minutes", ylab="Glucose in mmol/l")

        # FDA, first step: Curve fitting

library(fda)
mybasis         <- create.bspline.basis(rangeval=c(0,120),norder=4, breaks=c(0,30,60,90,120))            # Create B-spline basis (Appendix A)

        # Optimalisation of lambda, according to the generalised cross-validation criterion (Appendix A)

loglam          <- seq(-30,20,0.05)
# loglam        <- seq(0,20,0.05)              # Example of alternativ range for loglam in case of localt minimum (see below)
nlam            <- length(loglam)
dfsave          <- rep(NA,nlam)
gcvsave         <- rep(NA,nlam)

for (ilam in 1:nlam)
        {
        Lambda          <- 10^loglam[ilam]
        fdParobj        <- fdPar(mybasis,2,lambda)
        smoothlist      <- smooth.basis(c(0,30,60,90,120),t(g),fdParobj)
        dfsave[ilam]    <- smoothlist$df
        gcvsave[ilam]   <- sum(smoothlist$gcv)
        }
```

```
        # Optimal value of lambda:

lambdaopt      <- 10^loglam[gcvsave==min(gcvsave)]

        # Plot of gcv vs loglambda (ensure that the minimum is not a local one; if so, choose an alternative range for loglam, see above)
X11()
par(mfrow=c(2,3))
plot(loglam,gcvsave,type="l")
plot(loglam,gcvsave,type="l",ylim=c(0,100000))
plot(loglam,gcvsave,type="l",ylim=c(0,10000),xlim=c(-5,20))
plot(loglam,gcvsave,type="l",ylim=c(0,2000),xlim=c(-5,7))
plot(loglam,gcvsave,type="l",ylim=c(1200,1400),xlim=c(-5,-4))
abline(h=min(gcvsave),lty=2)
plot(loglam,gcvsave,type="l",ylim=c(1200,1400),xlim=c(2,4))
abline(h=min(gcvsave),lty=2)


        # Optimal smoothing, according to gcv criterion (Appendix A)

fdParobj.opt   <- fdPar(mybasis,2,lambdaopt)
g.smooth.opt   <- smooth.basis(c(0,30,60,90,120),t(g),fdParobj.opt)
plot(g.smooth.opt,lty=1,col="grey")

        # Plot of smoothed/fitted curves (Figure 1)

par(mfrow=c(1,2),mar=c(4,5,0,0)+0.2)
n=5
matplot(breaksuse,t(g[1:(n),]), type="b",pch= 20,lty=1, col=gray(0.85),lw=1,
        xlab="Time (min)", ylab="Glucose (mmol/l)", ylim=c(1.5,12),cex.lab=2,cex.axis=1.5,cex=2)
plot(g.smooth.opt$fd[1:n], lty=1, col=gray(0.5),lw=1,add=TRUE,
        xlab="Time (min)", ylab="", ylim=c(1.5,12),cex.lab=2,cex.axis=1.5)
plot(g.smooth.opt$fd[1:ng], lty=1, col=gray(0.5),lw=1,add=FALSE,
        xlab="Time (min)", ylab="",cex.lab=2,cex.axis=1.5)
lines(mean(g.smooth.opt$fd[1:ng]),col="black",lw=4)

        # Functional principal component analysis, FPCA (Appendix B)

g.pca          <- pca.fd(g.smooth.opt$fd,nharm=3)   # We keep only the first 3 FPCs
par(mfrow=c(1,3))
plot.pca.fd(g.pca)                                  # Quick FPCA plot
plot(g.pca$harmonics)                               # NB: 'harmonics' is another word for 'FPCA curves'

        # Bivariate correlations in Table 2

gdata                 <- cbind(g.pca$scores,g,(0.5*g[,1]+g[,2]+g[,3]+g[,4]+0.5*g[,5]),(g[,5]-g[,4]))
colnames(gdata)       <- c("fpc1","fpc2","fpc3","g0m","g30m","g60m","g90m","g2h","auc","shape")
head(gdata)
round(cor(gdata,use="pairwise.complete.obs"),2)
```

```
       # Nice FPCA plot (Figure 2 a-c)

eval.pca       <- seq(0,120,5)                          # The vector eval.pca makes sure that the +'s and -'s are not too close
g.pca.mean     <- eval.fd(eval.pca, g.pca$meanfd)       # Vector of mean values
g.pca.points   <- eval.fd(eval.pca, g.pca$harmonics)    # Vectors of harmonics-values (no. of vectors equals the no. of harmonics)
g.pca.values   <- g.pca$values                          # The variances of the score variables. Defines the 'multiplum' in Figure 2

ylim.g <- c(3,8.2)
par(mfcol=c(1,3), mar=c(2,4,1,1))
for(ii in 1:3){
plot(eval.pca,g.pca.mean, ylim=ylim.g, type="l",ylab=paste("Principal component curve",ii))
points(eval.pca,g.pca.mean + sqrt(g.pca.values[ii])*g.pca.points[,ii], pch="+",col="dark grey")
points(eval.pca,g.pca.mean - sqrt(g.pca.values[ii])*g.pca.points[,ii], pch="-",col="dark grey")
}

round(g.pca.values/sum(g.pca.values),3)                 # Percentage explained variance

       # Functional analysis of variance, FANOVA (Appendix C)
       # Data preparation (remove missing)

library(car)
dim(spssdata)
data           <-spssdata[!is.na(spssdata$g0m)&
                          !is.na(spssdata$g30m)&
                          !is.na(spssdata$g60m)&
                          !is.na(spssdata$g90m)&
                          !is.na(spssdata$g2h)&
                          !is.na(spssdata$bmi),]

g.bmi          <- cbind(data$g0m,data$g30m,data$g60m,data$g90m,data$g2h)
bmi.cut        <- cut(data$bmi,breaks=c(0,18.5,25,30,100), labels=FALSE, include.lowest=TRUE, right=FALSE)     # categorise into WHO categories
bmi.cut        <- as.factor(na.omit(bmi.cut))
bmi.cut        <- recode(bmi.cut,"1='6'")               # Make group 2 the reference category and code underweight as 6
table(bmi.cut)

bmi.smooth     <- smooth.basis(breaksuse,t(g.bmi),fdParobj.opt)
curves.bmi     <- bmi.smooth$fd

       # Mean glucose curves in the BMI categories (Fig. 4 a)

par(mfrow=c(1,2))
plot(mean.fd(curves.bmi[bmi.cut==6]),lw=4,ylim=c(3,8), col=gray(0.9),ylab="Glucose (mmol/l)",xlab="Time (min)",cex.lab=1.5)
plot(mean.fd(curves.bmi[bmi.cut==2]),lw=8,ylim=c(3.9,6.3), col=gray(0.8),add=TRUE )
plot(mean.fd(curves.bmi[bmi.cut==6]),lw=4,ylim=c(3.9,6.3), col=gray(0.9),add=TRUE)      # Plot underweight again to emphasise the crossing curves
plot(mean.fd(curves.bmi[bmi.cut==3]),lw=4,ylim=c(3.9,6.3), col=gray(0.5),add=TRUE )
plot(mean.fd(curves.bmi[bmi.cut==4]),lw=4,ylim=c(3.9,6.3), col=gray(0),add=TRUE )
```

```
m1.fanova             <- fRegress(curves.bmi ~ bmi.cut)           # The FANOVA
summary(m1.fanova$betaestlist)
X11()
par(mfrow=c(3,4))
lapply(m1.fanova$betaestlist, plot)

       # Plot of estimated beta curves only; a separate plot for the reference curve and an extra for the discrepancies (cf Fig. 4 b)

plot(m1.fanova$betaestlist$const,main="const")
plot(m1.fanova$betaestlist$bmi.cut.3,main="bmi 25-30",ylim=c(-1,1.5),col="red")
plot(m1.fanova$betaestlist$bmi.cut.4,main="bmi over 30",col="magenta",add=TRUE)
plot(m1.fanova$betaestlist$bmi.cut.6,main="bmi under 18.5",col="cyan",add=TRUE)

       # Plot of estimated beta curves with functional confidence intervals (editing not possible)

bmi.smooth.y2cMap     <- bmi.smooth$y2cMap
m1.fanova.yhat        <- m1.fanova$yhatfdobj$fd
m1.Errmat             <- t(g.bmi)-eval.fd(breaksuse,m1.fanova.yhat)
m1.SigmaE2            <- cov(t(m1.Errmat))
m1.fanova.std         <- fRegress.stderr(m1.fanova,bmi.smooth.y2cMap,m1.SigmaE2)

plotbeta(m1.fanova$betaestlist,m1.fanova.std$betastderrlist)

       # To make a plot like the one in Fig. 4 b, you need to make a modified version of the function plotbeta(); e.g. myplotbeta1()
       # Save it as an R script: myplotbeta1.R, and load it by

source("M:/ArticleFDA/myplotbeta1.R")
myplotbeta1(m1.fanova$betaestlist,m1.fanova.std$betastderrlist)    # (Figure 4 b)

       # Plots of dicrepancies between fitted curves and curves estimated from the model, as well as residuals

par(mfrow=c(2,2))
plot(curves.bmi,col=as.numeric(bmi.cut)+2,lty=1)
plot(m1.fanova$yhat$fd, lw=3,col="black",lty=1,add=TRUE)
yhatfd                <- m1.fanova$yhat$fd
plot(curves.bmi-yhatfd,col=as.numeric(bmi.cut)+2,lty=1)
res.matrix            <- eval.fd(breaksuse,curves.bmi)-eval.fd(breaksuse,yhatfd)
boxplot(t(res.matrix))

       # Permutation F tests for pairwise comparisons of BMI groups (Give the plots in Fig. 5)
       # Underweight vs normal weight: group 6 (bmi under 18.5) vs group 2 (Normal, bmi 18.5-25)

bmi.26         <- bmi.cut[(bmi.cut==2|bmi.cut==6)]
curves.bmi26   <- curves.bmi[(bmi.cut==2|bmi.cut==6)]
table(bmi.26)

cbasis         <- create.constant.basis(c(0,120))   # Make a constant basis
bmi.26         <- as.numeric(bmi.26)-1              # Recode factors "2" and "6" to the numbers 1 and 4; subtract 1 and get 0 and 3
bmi.26[bmi.26==3] <-1                               # Recode 3 to 1, i.e. 0 is the reference category and 1 is the category of interest
```

```
constfd        <- fd( matrix(1,1,length(bmi.26)),cbasis)
bmi26fd        <- fd( matrix(bmi.26,1,length(bmi.26)),cbasis)
xfdlist        <- list(constfd,bmi26fd )
betalist       <- list(fdParobj.opt,fdParobj.opt)

Fres12         <- Fperm.fd(curves.bmi26,xfdlist,betalist,nperm=1000)      # nperm should be large enough (here: 1000) to visualise the details
                                                                          # NB: Fperm.fd gives automatically a Permutation F Test plot
Fres12$pval
plot(Fres12$argvals,Fres12$pvals.pts, type="l")

       # Overweight vs normal: group 3 (bmi 25-30) vs group 2 (bmi 18.5-25)

bmi.23         <- bmi.cut[(bmi.cut==2|bmi.cut==3)]
curves.bmi23   <- curves.bmi[(bmi.cut==2|bmi.cut==3)]
table(bmi.23)
bmi.23         <- as.numeric(bmi.23)-1

constfd        <- fd( matrix(1,1,length(bmi.23)),cbasis)
bmi23fd        <- fd( matrix(bmi.23,1,length(bmi.23)),cbasis)
xfdlist        <- list(constfd,bmi23fd )
betalist       <- list(fdParobj.opt,fdParobj.opt)

Fres23         <- Fperm.fd(curves.bmi23,xfdlist,betalist,nperm=1000)
Fres23$pval
plot(Fres23$argvals,Fres23$pvals.pts, type="l")

       # Obese vs normal: group 4 vs group 2

bmi.24         <- bmi.cut[(bmi.cut==2|bmi.cut==4)]
curves.bmi24   <- curves.bmi[(bmi.cut==2|bmi.cut==4)]
table(bmi.24)
bmi.24  <- as.numeric(bmi.24)-1
bmi.24[bmi.24==2] <-1

constfd        <- fd( matrix(1,1,length(bmi.24)),cbasis)
bmi24fd        <- fd( matrix(bmi.24,1,length(bmi.24)),cbasis)
xfdlist        <- list(constfd,bmi24fd )
betalist       <- list(fdParobj.opt,fdParobj.opt)

Fres24         <- Fperm.fd(curves.bmi24,xfdlist,betalist,nperm=1000)
Fres24$pval
plot(Fres24$argvals,Fres24$pvals.pts, type="l")

       # Overweight vs obese: group 3 vs group 4

bmi.34         <- bmi.cut[(bmi.cut==3|bmi.cut==4)]
curves.bmi34   <- curves.bmi[(bmi.cut==3|bmi.cut==4)]
table(bmi.34)
bmi.34  <- as.numeric(bmi.34)-2
```

```
constfd        <- fd( matrix(1,1,length(bmi.34)),cbasis)
bmi34fd        <- fd( matrix(bmi.34,1,length(bmi.34)),cbasis)
xfdlist        <- list(constfd,bmi34fd )
betalist       <- list(fdParobj.opt,fdParobj.opt)

Fres34         <- Fperm.fd(curves.bmi34,xfdlist,betalist,nperm=1000)
Fres34$pval
plot(Fres34$argvals,Fres34$pvals.pts, type="l")

       # Analysing the effect of first trimester glucose curves on the 2-h value at wks 30-32 by multinomial regression
       # Data preparation (remove missing)

data    <- spssdata[   !is.na(spssdata$g0m)&
                       !is.na(spssdata$g30m)&
                       !is.na(spssdata$g60m)&
                       !is.na(spssdata$g90m)&
                       !is.na(spssdata$g2h)&
                       !is.na(spssdata$bmi)&
                       !is.na(spssdata$g2hwk30),]

       # Categorise into 7 categories

g.g2hwk30              <- cbind(data$g0m,data$g30m,data$g60m,data$g90m,data$g2h,data$bmi, data$g2hwk30)
g2hwk30.cut            <- cut(data$g2hwk30,breaks=c(0,3.27,3.89,6.39,6.90,7.8,8.84,100), labels=FALSE, include.lowest=TRUE, right=FALSE)
table(g2hwk30.cut)

g.g2hwk30.smooth       <- smooth.basis(breaksuse,t(g.g2hwk30[,-c(6,7)]),fdParobj.opt)
curves.g.g2hwk30       <- g.g2hwk30.smooth$fd

       # Mean glucose curves for groups of women in different glucose categories at gestational weeks 30-32 (Fig. 6)

plot(mean.fd(g.g2hwk30.smooth$fd[g2hwk30.cut==1]),  lw=4,ylim=c(2.5,7), col=gray(0.9),ylab="Glucose (mmol/l)",xlab="Time (min)",cex.lab=1.5)
plot(mean.fd(g.g2hwk30.smooth$fd[g2hwk30.cut==2]),  lw=4,ylim=c(3.9,6.4), col=gray(0.85),add=TRUE )
plot(mean.fd(g.g2hwk30.smooth$fd[g2hwk30.cut==3]),  lw=8,ylim=c(3.9,6.4), col=gray(0.8),add=TRUE )
plot(mean.fd(g.g2hwk30.smooth$fd[g2hwk30.cut==4]),  lw=4,ylim=c(3.9,6.4), col=gray(0.7),add=TRUE )
plot(mean.fd(g.g2hwk30.smooth$fd[g2hwk30.cut==5]),  lw=4,ylim=c(3.9,6.4), col=gray(0.55),add=TRUE )
plot(mean.fd(g.g2hwk30.smooth$fd[g2hwk30.cut==6]),  lw=4,ylim=c(3.9,6.4), col=gray(0.4),add=TRUE )
plot(mean.fd(g.g2hwk30.smooth$fd[g2hwk30.cut==7]),  lw=4,ylim=c(3.9,6.4), col=gray(0),add=TRUE )

       # FPCA of the reduced data set

g.g2hwk30.pca          <- pca.fd(g.g2hwk30.smooth$fd,nharm=3)
plot.pca.fd(g.g2hwk30.pca)
plot(g.g2hwk30.pca$harmonics)
g.g2hwk30.pca$values
round(g.g2hwk30.pca$values/sum(g.g2hwk30.pca$values),3)

mndata                 <- as.data.frame(na.omit(cbind(g.g2hwk30.pca$scores[,1:3],g2hwk30.cut,g.g2hwk30[,6])) )
colnames(mndata)       <- c("fpc1","fpc2","fpc3","g2hwk30kat","bmi")
mndata$g2hwk30kat      <- as.factor(mndata$g2hwk30kat)
```

```
       # Multinomial logistic regression

library(mlogit)
library(Epi)
mldata         <- mlogit.data(mndata, varying=NULL, choice="g2hwk30kat", shape="wide")
mref           <- mlogit(g2hwk30kat~1|fpc1+fpc2+fpc3+bmi, data = mldata, reflevel="3")
summary(mref)
exp(coef(mref))
mref           <- mlogit(g2hwk30kat~1|fpc1+fpc2+fpc3+bmi, data = mldata, reflevel="7")
summary(mref)
mref           <- mlogit(g2hwk30kat~1|fpc1+fpc2+fpc3+bmi, data = mldata, reflevel="6")
summary(mref)
mref           <- mlogit(g2hwk30kat~1|fpc1+fpc2+fpc3+bmi, data = mldata, reflevel="5")
summary(mref)
mref           <- mlogit(g2hwk30kat~1|fpc1+fpc2+fpc3+bmi, data = mldata, reflevel="4")
summary(mref)
mref           <- mlogit(g2hwk30kat~1|fpc1+fpc2+fpc3+bmi, data = mldata, reflevel="2")
summary(mref)

mref           <- mlogit(g2hwk30kat~1| g0m +bmi, data = mldata, reflevel="3")
summary(mref)
exp(coef(mref))
mref           <- mlogit(g2hwk30kat~1| g0m +bmi, data = mldata, reflevel="6")
summary(mref)
mref           <- mlogit(g2hwk30kat~1| g0m +bmi, data = mldata, reflevel="5")
summary(mref)
mref           <- mlogit(g2hwk30kat~1| g0m +bmi, data = mldata, reflevel="2")
summary(mref)

mref           <- mlogit(g2hwk30kat~1| g2h +bmi, data = mldata, reflevel="3")
summary(mref)
exp(coef(mref))
mref           <- mlogit(g2hwk30kat~1| g2h +bmi, data = mldata, reflevel="6")
summary(mref)
mref           <- mlogit(g2hwk30kat~1| g2h +bmi, data = mldata, reflevel="5")
summary(mref)
mref           <- mlogit(g2hwk30kat~1| g2h +bmi, data = mldata, reflevel="2")
summary(mref)

mref           <- mlogit(g2hwk30kat~1|shape+bmi, data = mldata, reflevel="3")
summary(mref)
exp(coef(mref))
mref           <- mlogit(g2hwk30kat~1|shape+bmi, data = mldata, reflevel="6")
summary(mref)
mref           <- mlogit(g2hwk30kat~1|shape+bmi, data = mldata, reflevel="5")
summary(mref)
mref           <- mlogit(g2hwk30kat~1|shape+bmi, data = mldata, reflevel="2")
summary(mref)
```
